# Perceived Barriers of Child Adoption: A Qualitative Study among Women with Infertility in Northern Ghana

**DOI:** 10.1155/2019/6140285

**Published:** 2019-06-09

**Authors:** Gilbert Ti-enkawol Nachinab, Ernestina S. Donkor, Florence Naab

**Affiliations:** ^1^Department of Midwifery, School of Allied Health Sciences, University of Development Studies, Tamale, Ghana; ^2^Department of Maternal and Child Health, School of Nursing, College of Health Sciences, University of Ghana, Ghana

## Abstract

**Background:**

Having a child is important among married women in Northern Ghana. Among married women, infertility is the main factor causing childlessness. Child adoption provides an alternative for married women to have children. The purpose of the study was to explore the perceived barriers of child adoption among women with infertility.

**Methods:**

The study used an exploratory qualitative approach to understand barriers of child adoption. The study was conducted among 15 women attending fertility clinic in a mission hospital in Northern Ghana. Participants were purposively recruited and data collected by individual face-to-face in-depth interviews. The interviews were audio-recorded, transcribed, and analysed using content analysis. Data were collected between January and March, 2016, in an office in the hospital.

**Results:**

The results suggest that barriers of child adoption include negative reaction of husbands, psychological dissatisfaction, and family dynamics. It was realised that husbands' reaction includes preference for biological children and marrying of second wives. Child adoption was psychologically dissatisfying to participants with some suggesting that it will make no difference and is a sign of acceptance of defeat in the quest to have biological children. The study findings also suggested that family dynamics that could hinder the practice of child adoption includes high value for blood relations, blaming of the woman, unpredictable family influence, discrimination against the adopted child, and family not allowing the adopted child to inherit property.

**Conclusion:**

The preference for biological children is by far an outstanding barrier and a major influence of all the emanating barriers associated with child adoption. There is the need for public education and special counselling session for husbands and other family members on child adoption as an alternative solution for infertility.

## 1. Introduction

Infertility is a reproductive disorder and a global public health problem that can affect both men and women and has no predilection for national, ethnic, racial, and religious background [1]. Infertility is the inability to attain a successful pregnancy after at least 12 months of unprotected or more of appropriate timed unprotected intercourse or therapeutic donor insemination [2]. Infertility is a global problem affecting between 50 million and 80 million people translating into 8% to 12% of couples worldwide [3]. In Africa, studies suggest that the burden of infertility is about 30–40% [3]. The WHO has classified infertility into two categories: primary and secondary. Primary infertility is the inability to bear a child in a couple who have never had a child after more than one year of unprotected intercourse, while secondary infertility is the failure to bear a child which can be a result of being unable to become pregnant or carry a pregnancy to live birth after having a child [4].

In a typical African society, people normally start a family by getting married and giving birth to their own children. Childbirth is an expectation of marriage; hence couple who are unable to achieve this come under intense pressure to conceive and this increases their anxiety significantly regardless of their educational or economic status [5, 6]. For women with infertility who are of rural background and lower income status, finding medical help could be challenging. Child adoption could therefore be an alternative for women with infertility who desire children [7, 8].

Child adoption started as a way to preserve family lineage, secure inheritance, and forge alliances but has progressively evolved into a process for meeting the needs of adults who need children of their own [9]. In Ghana, there are two forms of child adoption which include customary adoption and formal adoption. Customary adoption is an informal transfer of the care of a child to another relative. In the formal adoption process, legal requirements must be met and a formal procedure followed. The requirements and process of formal adoption are contained in the 1998 Children's Act [10]. In some situations health personal department encourages people to adopt children that are abandoned in hospitals. Children adopted this way are not captured in the government statistics making it quite difficult report accurately on the rates of child in Ghana [11].

One key benefit that comes with child adoption is the provision of an alternative for having children by couples suffering from infertility [12–14]. Child adoption could bring joy and fulfillment to couples with infertility [15]. In other African societies, most issues that will lead to child adoption include infertility and desire for heir for a lineage [14].

In Africa, child adoption is gradually becoming known in most countries but culture is posing a seeming setback to its acceptability and practice among various ethnic groups [16–18]. Aside from cultural implications, studies have also pointed to other militating factors against child adoption such as misconceptions, stigmatization, financial burden, and procedural bottlenecks [12, 19].

Certain deeply ingrained traditional beliefs tend to influence societal attitude towards child adoption [16]. For instance, in the Yoruba land of South-Western Nigeria the belief that “*ori omo lo npe omo wa'ye*” which means an adopted child usually attracts yet to be born children to come to the physical realm could positively influence the practice of child adoption [8]. In some communities, both the adopted child and the adoptive parents are stigmatized because the cultural belief considers the child as a second best and the parents as being unrelated to the child genetically [15, 20, 21].

Cost of child adoption and the waiting period are notable challenges of formal child adoption in Ghana. In some cases prospective adoptive parents will have to wait for up to years [11]. Though child adoption can help meet the need of couples for a child, there is dearth of studies on the associated factors such as attitude and perception on the uptake of child adoption in Ghana. The purpose of this study was therefore to explore the barriers of child adoption among women with infertility.

## 2. Methods

### 2.1. Design

A qualitative approach helps in revealing subjective realities and truths about the meaning and expressions of participants [22]. The study used an exploratory qualitative approach to explore the perceived barriers of child adoption. This allowed the researchers to follow up on emerging views on the perceived barriers of child adoption.

### 2.2. Study Setting

The study was carried out in a Mission Hospital Northern Ghana. The hospital has a permanent obstetrician/gynaecologist and operates a fertility clinic which enabled the researchers to have access to women with infertility.

### 2.3. Target Population and Sampling Technique

Selection of participants in qualitative research is normally based on unique knowledge, experiences, or views of the participants on the subject under investigation [23]. The study targeted women who have been married for at least a year, engaged in unprotected sexual intercourse, and not using contraceptives yet unable to become pregnant. They were also women who could speak Kusaal, Mossi, or English language. Purposive sampling technique was used to select participants who met the inclusion criteria. Data saturation (a point at which no new information emerges from the interviews) was reached by the time 15 women were interviewed.

### 2.4. Data Collection Tool

Data were collected using a semistructured interview guide which was developed by the researchers based on the objective of the study. The participants were asked questions such as “what are some of the things that will make it difficult for you to practice child adoption? What will be your husbands' position if you suggest child adoption to him? What will be the reaction of your family members if you decide to adopt a child? How do you think an adopted child will be treated in your family and community?”

### 2.5. Data Collection Procedure

The principal investigator obtained formal permission from the authorities of the Mission Hospital. Two nurses working in the consulting room of the obstetrician/gynaecologist first contacted and explained the study to potential participants and those who agreed were referred to the principal investigator in a separate room in the facility. The participants were then further screened to ensure that they met the inclusion criteria and once they voluntarily accept to participate, the time and date was scheduled for data collection. Depending on participants' preference, some interviews were done on the same day of contacting the participants while others were on subsequent visits. Data were collected between January and March, 2016, in an office in the hospital. Each interview lasted 35-45 minutes and with participants' permission the interviews were audio-recorded and field notes taken. The interviews were conducted face to face in Mossi, Kusaal, Frafra, or English Language. The interviews that were done in Kusaal and Mossi were transcribed in English based on the meaning of the statements. The transcripts were discussed with an expert in Kusaal and Mossi language and confidentiality was ensured in the process.

### 2.6. Data Analysis

The data was analysed using Thematic Content Analysis (TCA). TCA is descriptive presentation of data and the most foundational of qualitative data analysis. TCA allows researcher to peruse and group the entire textural data into a list of common themes that gives a voice or true representation of the entire data set [24]. The audio-recorded interviews were transcribed verbatim taking into consideration field notes. The transcripts were read severally by the researchers to understand the world of the participants regarding the issues raised. The key ideas in the form of phrases, sentences, or paragraphs emerging were assigned codes. Labels for initial codes containing several thoughts emerged. These codes were sorted into themes based on how they were related and linked. Each of the three researchers coded the data individually and several group discussions were held to agree on main themes and emerging subthemes. The main themes and subthemes were revised repeatedly until being suitable for presentation of findings. The data was managed manually.

### 2.7. Ethical Consideration

Ethical clearance for a broader study from which this paper is drawn was obtained from Ethics Review Board of Noguchi Memorial Institute for Medical Research, University of Ghana (NMIMR-IRB CPN 013/15-16). Participants gave informed consent to participate in the study. Participants were informed that participation was voluntary. Anonymity was ensured by labelling each participant's information with a pseudonym. Pseudonyms used were names in languages spoken in southern part of Ghana; hence none of the names in the transcribed data is related to any of participants.

### 2.8. Methodological Rigour

“Rigour of a qualitative study is the extent to which the identified meanings represent the perspectives of the participants accurately” [25, pp. 68]. It can be argued that without rigour the research conducted can become fictional and worthless in adding knowledge [26]. According to Grove et al. [25], the degree of rigor of a study findings is determined by the extent to which the findings are credible, transferable, dependable, and confirmable. The four criteria for ensuring rigour according to Guba [27] were followed.

#### 2.8.1. Dependability

Dependability refers to the study yielding similar results with other groups in a similar context [28]. To ensure dependability, the method used for data collection and analysis is captured in the report. Nonparticipants with similar experiences were also contacted to validate findings of this study.

#### 2.8.2. Confirmability

This refers to the extent to which other researchers can review the audit trail and agree that the authors' conclusions are logical [29]. To ensure this the researchers kept an audit trail comprising of field notes, audio recordings, analysis notes, and coding details.

#### 2.8.3. Credibility

This involves the confidence of the reader about the extent to which the researcher has produced results that reflects the participants view [29]. The researchers conducted a face-to-face interview to enable probing and getting more important information. Data was audio-recorded and transcribed verbatim taking into consideration field notes. To ensure that the study findings were credible a member check was done by tracing some of the participants to confirm the accuracy of transcribed data and emerging themes.

#### 2.8.4. Transferability

This refers to the ability to move the findings of the qualitative study to other contexts with similar groups [30]. Transferability was ensured through thick description which involves a rich and thorough description of the research setting, the context where interviews were conducted, and the processes throughout the investigation.

## 3. Results

### 3.1. Demographic Characteristics of Participants

Table 1 shows that the study participants were 15 married women between the ages of 24 and 40 years. Nine of them had primary infertility and the rest had secondary infertility. Among the participants with secondary infertility, four of them had one child each, another had two children, and the other had three children. Five participants were in polygamous marriage. Ten of the participants were Muslims and five were Christians.

Table 2 shows a thematic presentation of the findings which include three major themes with their corresponding subthemes

### 3.2. Negative Reaction of Husbands

All women recognized the role of their husbands in taking a decision like child adoption. The women had different reasons why they thought their husbands would not agree to practice child adoption. Reasons for not accepting child adoption include marrying a second wife and preference for a biological child

### 3.3. Husbands Prefer Marrying a Second Wife

According to the women, their husbands perceive that marrying a second wife will increase their chances of getting a child. Husbands having children or hoping to have children with a second wife was a reason why husbands will not accept child adoption. Child bearing in marriage perhaps was seen as something to satisfy the need for a biological child by the husband.

“For my husband he will not agree. He has children with my rival so he will not agree to bring in a child that is not his. If he had no child and I was the only wife, maybe we could talk about it but as it stands he will just disagree” (Adwoa).

“My husband has married another wife so he will not agree for us to adopt a child because he knows that my rival will start having children soon” (Serwa).

### 3.4. Husbands Prefer a Biological Child

In Northern Ghana, children normally bear names of the father or the family as their surname and this is exclusively reserved for biological children. Some participants also explained that their husbands will prefer their own biological because of preference for blood relations. With this perception, the participants felt that their husbands will not see adopting a child as a solution to their childlessness.

“My husband will not agree because he will think the adopted child does not have his blood. In a family here, they want their own blood; if not they will not see you as part of them” (Asantewa).

Husbands approval which is vital in child adoption was seen by some participants as a major obstacle.

“It will be difficult to convince my husband. It will take him time to accept the idea. This is because of denial which I think is normal. Everyone wants his own child and men are more serious with that” (Yaa).

### 3.5. Psychologically Dissatisfaction

Psychological dissatisfaction was seen as one of the most notable barriers to child adoption. The women explained that adopting a child will not come with the required psychological satisfaction associated with having a biological child and will even indicate an acceptance of the plight of infertility.

### 3.6. Adopting a Child Will Not Make Any Difference

Some participants thought that they simply need their own children while others thought that adopting will not let the family recognise them as people with biological children.

“Nothing will change in my life if I adopt a child. You will feel like you have a child but deep down your heart it will not feel like it is your own child. The people in the family will not see you as somebody with her own child” (Adwoa).

“Adopting a child will only be like trying to clean your tears but this will not relieve the pain of childlessness” (Afia).

### 3.7. Adopting a Child Is a Sign of Accepting Defeat

The women presence at the fertility clinic was strongly backed by the hope that they will find a solution to the problem of infertility. Adopting a child was therefore seen as creating an impression that one had been told that you will never have your own children.

“Well, adopting a child will not satisfy me in my mind that I have delivered. People will look at you and think you have been told that you can no longer deliver and that is why you are bringing a child to satisfy yourself” (Serwa).

“You can only adopt if only you know you will not be able to get pregnant at all. If not, you have to wait till you get pregnant. As a Christian I have faith that no matter how long it takes I will get pregnant” (Ohema).

### 3.8. Family Dynamics

Family influence plays an important role when it comes to child adoption. Family dynamics which was one of the main themes described the high value placed on blood relations, blaming of the woman, unpredictable family influence, discrimination against the adopted child, and family not allowing the adopted child to inherit property.

### 3.9. High Value on Blood Relations

Most of the participants explained that their families will not accept the adopted child because of the high value placed on blood relations.

“Their (referring to the husband) tribe will not accept a child that is not carrying their blood. They will refer you to others who are not having their own but have not adopted a child” (Boatema).

“The family will not treat an adopted child well because they like blood relations. They will see the child to be an alien and not part of them. The way people will treat the child you will be having problems with them always” (Afia).

### 3.10. Blaming the Women

Our participants explained that the woman is often blamed for the situation of childlessness in the marriage. To solve the problem, the family will suggest that the man marries more wives in an attempt to increase his chances of getting a child.

“In Islam a man can marry more than one wife so the family will prefer my husband going for a second wife. This is because the local people never think that the problem could also be coming from the man. They always think it is the woman. So if you are not conceiving another wife has to come” (Agyeiwa).

“Just look at this clinic and you will realise that we are all women. Our husbands are at home and we are here trying to solve the problem alone. They think it is only the woman who has a problem” (Afia).

### 3.11. Unpredictable Family Influence

It was underscored that inability to attain childbirth is an issue of concern to the family of the affected person and seeking solution is often a collective responsibility. In this direction, family members may make suggestions as to where to obtain help in achieving conception. The women were of the view that adopting a child will demand their husbands discussing it with family members and this makes it even more difficult to what the reaction will be.

“That one I can't tell what my husband will say. He will like to discuss it with his family members and with that it will even be difficult to predict what will happen” (Fosuah).

### 3.12. Discrimination against the Adopted Child

Some participants thought the family and community at large will not treat the child well. The adopted child will receive insults and this will hurt the adoptive parents as well.

“……………..if it is a boy who has to be a member of the family without leaving, there will be problems. The family will not treat the child well. They will insult him “sankpase” (bastard) especially if he is a boy. You will always have problems with them because of how they will treat the child” (Akua).

Participants also explained that adopting a child will attract negative comments from community members towards both the woman and the adopted child.

“The child will suffer in our community because they will keep calling the child “tampiire” (a person without father). Even when a woman delivers out of wedlock and has to leave that child with her biological parents, they will be insulting the child like it is the child's fault. An adopted child will suffer worse insults” (Yaa).

### 3.13. The Family Will Not Allow the Adopted Child to Inherit Property

The adopted child will also suffer future uncertainties such as denial of the opportunity to inherit the adoptive parents' property. The decision regarding legitimacy to inherit property is taken by the family and is normally given to biological male children.

“On the part of my husband's family, it is going to be a problem if you adopt a child. Not now but years to come. They will tell you all sort of things and the child will have problems in the future especially with property. They will be using that one against you so that the child will know he is not their blood and should not come near anything belonging to the man” (Boatema).

“After the death of you, the parents, they will not allow the adopted child to inherit your property. That is when they will start saying “you are a bastard, go to your father or go and find your parents.” To let such a poor child go through this kind of treatment, it is better I stay away from adopting a child” (Agyeiwa).

## 4. Discussion

The study explored the perceived barriers of child adoption as an alternative solution for infertility. The study found that women considered a decision to adopt a child as a very important one that centres on the approval of the husband. In Northern Ghanaian society, the man is considered the head of his nuclear family and takes all the important decisions on behalf of the family.

The study finding suggests that polygamy is used by husbands in an attempt to get biological children rather than seeking treatment together with the first wife. This concurs with the study findings that women with infertility are most likely to find themselves in polygamous marriages because their husbands are encouraged by relatives to marry second wives so as to produce children for continuity of the family [31]. Perceived negative reaction of husbands towards child adoption was based on the desire or presence of a second wife and this can be connected to the religious background of the couple. This is because polygamy is allowed in the Islamic religion; thus in this study all those in polygamous marriages were Muslims.

Delivering a child in every marriage is often seen as an important achievement and a sign that the marital relationship is full of blessings and thus psychologically satisfying to the couples. The women indicated that there was a psychological dichotomy between having a biological child and getting a child by adoption. This finding concurs with that of a study in Iran which cited psychological dissatisfaction as a militating factor against child adoption [32]. Another important psychological dissatisfaction reported in our study was that some women thought that adopting a child will create an impression that you have lost the struggle to get your own child. This stance seems to agree with the Christian maxim that “with God all things are possible” which inspire followers that they should never give up in any situation in life because God will answer them in due course. This urges them on to continue struggling for their own children rather than going in for child adoption

The study findings show that there is high premium on biological parenting. In northern Ghanaian society a family is defined by how the members are related by blood or through marriage. A person who is not connected to a family by blood or marriage is there not considered a member of the family. The participants were generally aware of this stand and thought that one will have problems if you try to bring adopted child into family. They further indicated that the child will not enjoy any sense of belonging in the family and will be treated as an alien. Similarly, a study in South-Eastern Nigeria established that child adoption was not accepted there because the culture does not create room for the adopted child to step into the shoes of adoptive parents as if he is a biological child [17].

The study findings suggest that women are blamed for the childlessness situation in the marriage putting them under pressure to make efforts to ensure that they get pregnant. This position of the family could result in the family urging the man to marry another wife if the woman fails to get pregnant. The assumption that the woman is responsible for childlessness shows that there is little literacy on the causes of infertility and the fact that it could affect men as well. Another evidence of the blame of childlessness being put on the woman was by the fact that the fertility clinic was attended by only women. This is in consonance with the study finding that women are those who mostly report at fertility centres and undergo most of the invasive procedures making them bare greater part of the psychological distress associated with infertility [33].

The participants indicated that adopting a child was a major decision that could receive unpredictable family influence. They pointed out that the decision to adopt a child will not rest entirely on their husbands but the family as well. In the Ghanaian context, the coming of a child into a family is often regarded as an important event; hence child adoption which makes the child a permanent member of the family will demand consent from the family members. The husband and wife are thus not considered independent to take the decision to adopt a child. Sami and Ali [34] reported that the decision to consider child adoption does not only depend on husbands but depends on other family members as well. In typical Ghanaian tradition, child upbringing is considered a responsibility of the entire family and hence the need to consult and agree with family before child adoption.

The findings indicated that an adopted child will not be treated well but will rather suffer discrimination. Participants of the Kusasi tribe stated that the child will suffer a lot of insults and may even be referred to as “tampiire” (a person without known biological parents) or “sankpase” (bastard). Similarly, the unacceptability of child adoption in Eastern Nigeria is influenced by the indigenous ideology of “onyebiaraabia” which means “the stranger” [16]. In an attempt to protect the child from being insulted, the participants viewed that the adoptive parents will constantly have problems with other family or community members.

In Northern Ghanaian society, property of a man is traditionally inherited by the first born male who then shares with his male siblings. The study findings indicate that the adopted child will not be allowed to inherit or share the property of the adoptive parents. The participants find this denial of inheritance so humiliating that they will rather stay away from adopting a child who will obviously suffer in the future.

## 5. Conclusion

This study suggested a number of barriers to child adoption but central among them is the high premium placed on biological parenting. The preference for a biological child is influenced by cultural and religious background of the people. Though women suffering infertility go through a lot in an attempt to find a solution, the decision to adopt a child largely depends on the husband and his family. Awareness creation on the viability of child adoption as an alternative solution for infertility should therefore involve husbands, key family members, and religious leaders in the community.

Also, the study findings indicate that women in childless marriages are blamed for the situation. Women are under pressure to find a solution to the childless situation while their husbands are encouraged to marry again to increase their chances of getting a child. This shows lack of knowledge on causes of infertility. There is the need to educate the public on the causes of infertility and to let people understand that infertility could occur in any of the genders.

### 5.1. Limitations of the Study

- The study is the first to explore the barriers of child adoption as an alternative solution for infertility in Ghana. Hence, other studies can be done to gain a holistic picture of the barriers and what can be done to facilitate the utilistaion of child adoption.
- The study did not include husbands of the women so that the ideas on barriers could represent both husbands and wives as an entity.
- The small size of the study participants does not make the study findings generalizable to other settings in Ghana.

## Figures and Tables

**Table 1:** Sociodemographic characteristics of participants.

Pseudonym	Age	Level of education	Number of wives of the husband	Years of marriage	No. of children	Occupation	Years of searching for a child	Religion
Abena	28	None	1	13	3	Housewife	4	Muslim
Adwoa	40	None	2	16	None	Farming	16	Muslim
Akosua	29	None	1	10	2	Trading	2	Muslim
Ama	26	Tertiary	1	5	None	Teacher	5	Muslim
Afia	34	Primary	2	12	None	Trader	10	Muslim
Akua	30	Secondary	1	7	1	Trader	3	Christian
Agyeiwaa	24	Tertiary	1	13 months	None	Teacher	1	Muslim
Boatemaa	25	Tertiary	2	2	None	Teacher	2	Muslim
Araba	31	None	1	4	None	Housewife	4	Muslim
Asantewaa	30	None	1	5	None	Housewife	3	Muslim
Fosuaah	27	Tertiary	1	6	1	Nursing	2	Christian
Ohema	37	Tertiary	1	9	None	Civil servant	8	Christian
Serwa	34	None	2	7	1	Housewife	5	Muslim
Owusuaa	31	None	1	5	None	Civil servant	4	Christian
Yaa	28	Tertiary	1	6	1	Nursing	2	Christian

**Table 2:** Main themes and subthemes.

Main themes	Subthemes
Negative reaction of husbands	Husbands prefer marrying second wife
	Husbands prefer biological children
Psychological dissatisfaction	Adopting a child will not make any difference
	Child adoption is a sign of accepting defeat
Family dynamics	High value for blood relations
	Blaming of the woman
	Unpredictable family influence
	Discrimination against the adopted child
	The family will not allow the adopted child to inherit property

## Data Availability

The original transcripts from which the paper emanated are available upon request.
